# Machine learning and expression analyses reveal circadian clock features predictive of anxiety

**DOI:** 10.1038/s41598-022-09421-4

**Published:** 2022-04-01

**Authors:** Aziz Zafar, Rebeccah Overton, Ziad Attia, Ahmet Ay, Krista Ingram

**Affiliations:** Department of Biology, Colgate University, 13 Oak Drive, Hamilton, NY 13346 USA

**Keywords:** Circadian rhythms and sleep, Neuroscience, Anxiety, Depression, Behavioural genetics, Gene expression, Genetic association study

## Abstract

Mood disorders, including generalized anxiety disorder, are associated with disruptions in circadian rhythms and are linked to polymorphisms in circadian clock genes. Molecular mechanisms underlying these connections may be direct—via transcriptional activity of clock genes on downstream mood pathways in the brain, or indirect—via clock gene influences on the phase and amplitude of circadian rhythms which, in turn, modulate physiological processes influencing mood. Employing machine learning combined with statistical approaches, we explored clock genotype combinations that predict risk for anxiety symptoms in a deeply phenotyped population. We identified multiple novel circadian genotypes predictive of anxiety, with the *PER3*(rs17031614)-AG/*CRY1*(rs2287161)-CG genotype being the strongest predictor of anxiety risk, particularly in males. Molecular chronotyping, using clock gene expression oscillations, revealed that advanced circadian phase and robust circadian amplitudes are associated with high levels of anxiety symptoms. Further analyses revealed that individuals with advanced phases and pronounced circadian misalignment were at higher risk for severe anxiety symptoms. Our results support both direct and indirect influences of clock gene variants on mood: while sex-specific clock genotype combinations predictive of anxiety symptoms suggest direct effects on mood pathways, the mediation of *PER3* effects on anxiety via diurnal preference measures and the association of circadian phase with anxiety symptoms provide evidence for indirect effects of the molecular clockwork on mood. Unraveling the complex molecular mechanisms underlying the links between circadian physiology and mood is essential to identifying the core clock genes to target in future functional studies, thereby advancing the development of non-invasive treatments for anxiety-related disorders.

## Introduction

Mood disorders, including depression and anxiety, are becoming more prevalent globally, affecting nearly one-fifth of the adult population^1^. These disorders negatively impact productivity, social relationships, and overall quality of life in individuals. The search for genetic and environmental factors contributing to the epidemic of mental health has uncovered numerous links between circadian rhythm disruptions and mood disorders including major depressive disorder (MDD), schizophrenia, bipolar disorder (BD), and generalized anxiety disorder^2–7^. Clinical studies have also demonstrated that circadian rhythms and circadian clock genes can modulate mood and psychiatric disorders but few of these studies have explicitly focused on anxiety^8^.

Circadian rhythms regulate a sleep–wake cycle that is reset every 24 h based on exposure to natural or artificial light–dark cycles. The molecular clock driving these rhythms is created by feedback loops in core clock genes and their associated transcription factors that control physiological cycles in the body via the regulation of over a third of all transcribed genes^9,10^. The core feedback loop includes the transcription factor CLOCK, which regulates the transcription of genes in the Period (*per1, per2*, and *per3*) and Cryptochrome (*cry1* and *cry2*) gene families. The CLOCK protein forms a heterodimer with BMAL1, which activates additional core clock genes. PER/CRY heterodimers, in turn, inhibit the activity of the BMAL1-CLOCK complex. This cycle of transcription activation and repression is vital for the 24-h circadian cycle. Mutations in these core clock genes may affect mood via direct transcriptional activity of downstream physiological pathways that influence mood. Alternatively, clock gene mutations may modulate mood pathways indirectly—through disruptions in the phase and amplitude of circadian rhythms^11^. Individual core clock genes may, in fact, be involved in both direct and indirect mechanisms modulating the relationship between mood and circadian rhythm.

Evidence supporting indirect impacts of the core circadian oscillator on mood pathways is derived from studies examining the association of diurnal preference, differences in timing of activity levels, or chronotype, differences in sleep–wake timing, with mood^7,12–16^. Individuals that are morning types tend to have earlier circadian phases and sleep–wake rhythms than the intermediate and evening types. These behavioral patterns parallel physiological changes, such as changes in body temperature and melatonin profiles^17^ and clock gene expression oscillations (molecular chronotypes) associated with diurnal preference^5,13,15,18–21^. Studies on diurnal preference show that evening types are more likely to experience depression or anxiety at one point in their lifetime^17,22–29^. Mood disorders have also been linked to diurnal preference and circadian misalignment—a mismatch between an individual’s physiological circadian rhythms and their behavioral cycles^10,16^. Previous genome-wide association studies (GWAS) and candidate gene studies have also found associations between multiple circadian genes and diurnal preference^7,14^ as well as depression or other psychological disorders^13^. These studies provide strong evidence that diurnal variation in circadian rhythms plays a role in modulating the physiology of mood disorders. However, attempts to determine the molecular mechanisms underlying these indirect circadian influences on mood and anxiety, in particular, are in a nascent stage.

Core circadian genes also function as transcriptional regulators and can influence neurotransmitter signaling in well-known mood pathways, including serotonin, dopamine, and glucocorticoid pathways^30^. Recent studies demonstrate that components of the circadian clock can directly modulate mood disorders. In mice, knock down of the *Neuronal PAS Domain Protein 2 (NPAS2), period (PER),* or *cryptochrome* (*CRY*) genes results in altered anxiety levels^9,31,32^. In humans, genome- and phenotype-wide association studies (GWAS and PheWAS) have not yielded strong evidence of associations between core clock genes and mood until Ho et al.^33^ identified a clock-related gene associated with seasonal affective disorder. However, population-level candidate gene studies have identified multiple links between clock genes and depressive disorders, including anxiety^3,7,32^. Mathematical models have also predicted links between circadian clock disruptions and anxiety^14^. Most interestingly, a recent clinical study demonstrated that melatonin treatment used to correct circadian misalignment in anxious patients helped to mitigate anxiety^34^. The lack of clarity in large GWAS and PheWAS studies with complex phenotypes like mood disorders suggests that directed candidate gene studies using deep phenotyping are needed to identify the influence of specific clock genes and/or synergistic clock gene interactions on mood pathways.

In the current study, we performed a deep phenotypic analysis of anxiety (State-Trait Anxiety Inventory (STAI))^35^ diurnal preference (Morningness–Eveningness Questionnaire (MEQ)^36^, and molecular chronotype (via gene expression analyses based on clock gene expression phase and amplitude). We explore the synergistic effects of multiple genotypes on phenotypes using an array of machine learning algorithms, including feature selection and association rule learning, as well as statistical approaches. Unlike PheWAS studies, machine learning techniques are not constrained by and can be robust to the smaller sample sizes typical of deeply phenotyped datasets. In addition, feature selection can be used to incorporate both clinical and genotypic features to reduce the data’s dimensionality and identify the most predictive disease risk factors. Here, we employ deep-phenotyping and machine learning to identify direct and indirect mechanisms of circadian influences on anxiety.

## Methods

### Experimental data collection

Study participants were recruited from Colgate University and the surrounding community in Hamilton, NY, USA (n = 982; males = 318, females = 664, ages 17–79; median = 19). Participants were predominantly Caucasians of European descent. All participants gave written informed consent, and all procedures followed the principles of the Declaration of Helsinki. The Institutional Review Board at Colgate University authorized all consent forms and procedures (#FR-F13-07, #ER-F14-12, #F15-13, and #ER-F16-19).

### Self-report surveys

Participants completed computer-based surveys, which included the trait version of the Spielberger’s State-Trait Anxiety Scale (STAI)^37^, Beck Depression Inventory (BDI-II)^38^, and the short form of the Patient-Reported Outcomes Measurement Information System (PROMIS™)^39^ Sleep Disturbance. The STAI was used to indicate the anxiety scores of individuals ranging from 20 to 80, with scores of 20–37 indicating “no or low anxiety”, 38–44 indicating “moderate anxiety”, and 45–80 indicating “high anxiety.” The Horne-Östberg Morningness-Eveningness Questionnaire (MEQ)^36^ survey was administered to measure diurnal preference.

### Genotyping

DNA was extracted from 10 to 20 hair follicles from each participant. The hair samples were digested with Proteinase K at 56 °C for 24 h, and purified using the Qiagen DNAeasy Micro Kit. Genotyping for single nucleotide polymorphisms (SNPs) was performed using a TaqMan SNP Genotyping assay (Applied Biosystems, Foster City, CA) on an ABI 3700HT real-time qPCR instrument. Participants were identified as homozygous or heterozygous for the major and minor alleles (Suppl. Table 1).

A fragment length analysis of the *PER3* VNTR length polymorphism repeat region was conducted using PCR fluorescent primers on GeneScan software with an ABI 3100 sequencer. The forward primer fluorescently labeled with 6-FAM was used with the following PCR primers: forward, 5′-CAAAATTTTA TGACACTACCAGAATGGCTGAC-3′, and reverse, 5′-AACC TTGTACTTCCACATCAGTGCCTGG-3′^40^. The PCR was performed in a 25-μl volume using Qiagen PCR Mastermix. The PCR cycling conditions were 3 min at 94 °C, followed by 35 cycles of 45 s at 94 °C, 45 s at 58 °C, and 45 s at 72 °C, with a final step at 72 °C for 3 min. Capillary electrophoresis was then used to separate *PER3* alleles on an ABI 3700 sequencer and sized using ABI ROX standards. The genotype of each participant was identified as *PER3* 4/4, *PER3* 4/5, or *PER3* 5/5.

### Circadian gene expression analysis (molecular chronotyping)

Ten to twenty hair follicles were collected in RNAlater solution at four different time points during the day: 8 a.m., 4 p.m., 5 p.m., and 8 p.m.^16^. All hair samples were stored at − 80 °C prior to analysis. RNA was extracted and purified from hair follicles using the RNeasy Micro purification kit according to the protocol provided by Qiagen. The purified RNA was converted to cDNA using rt-PCR (TaqMan Gold rt-PCR, ABI). Nanodrop was used to quantify the cDNA. Expression levels of clock genes *PER3* and *NR1D2* were measured using quantitative PCR on an ABI 7900HT instrument (Applied Biosystems). *GUSB* and *18S* were used as control genes, and each analysis was performed in a replicate of three. Relative mRNA levels were determined using the standard curve method as described in the ABI User Bulletin #2, and then converted into z-scores per individual. A standard curve was created based on the average data points from all the subjects^41^. The trained curve was fitted to the four data points of each subject using the parameter estimation method, Stochastic Ranking Evolutionary Strategy (SRES). 2 For phase shift estimation, the training curve was obtained from known intermediate types (n = 20; individuals not included in this study). The phase difference between the curve obtained from each subject’s four RNA data points and the training curve gave the phase shift. Amplitudes and phases were then converted into z-scores for statistical analysis.

### Feature generation and selection

#### Genotypic and clinical features

We used seven genotypic features: *CLOCK*3111 (rs1801260), *CRY1* (rs228716), *CRY2* (rs10838524), *PER2* (rs10838524), *PER3A* (rs228697), *PER3B* (rs17031614), and *PER3* VNTR (rs57875989), and four behavioral/clinical features: diurnal preference scores, age (≤ 22 or > 22), gender, and socioeconomic status (poor, lower-middle-class, upper-middle-class, affluent). For genotypic features, individuals can be homozygous dominant, homozygous recessive, or heterozygous. To reduce multicollinearity, we performed one-hot encoding for non-binary features, removing the most frequent variant (as the baseline condition). We created 2-way combinations using the seven genotypic features and their respective variants, giving us 7C2 * 9 more features. We removed the most frequent class for each group of 9 combinations and treated it as a reference category. After pre-processing, the data set for analysis contained 174 total features.

#### Feature selection

We used four feature selection methods (each method ten times with ten-fold cross-validation) to find epistatic combinations predictive of mood disorders. InfoGain (IG) and ReliefF (ReF) are ranking-based feature selection methods that rank features based on their correlation with the class^42,43^. Minimum Redundancy Maximum Relevance (MRMR) and Joint Mutual Information (JMI) are subset-based feature selection methods that use information theory-based criteria to find possible subsets from the feature space^44,45^. A feature was considered robust if it appeared in 95% of the runs for a certain feature selection method and in at least three out of the four feature selection methods. This subset of the features was used for in-depth statistical analysis.

#### Classifiers

We modeled the relationship between risk factors and anxiety using three classifiers: tree-based methods like Random Forests (RF) and XGBoost (XGB) and a linear method, Support Vector Machines (SVM)^46–48^. We evaluated the performance of our classifiers using accuracy scores and the area under the receiver operating characteristic (AUROC) curves. Classifiers employ a variety of hyperparameters that must be tailored for each dataset. As a result, we used preliminary testing to determine an appropriate range of hyperparameters for each classifier, followed by grid searching to determine the optimal combination of hyperparameters for maximizing accuracy.

#### Cross-validation

We employed stratified tenfold cross-validation to determine each model’s generalizability. We divided the data set into tenfolds(subsets) for each combination of feature selection method and classifier, maintaining a consistent distribution of our outcome class for each fold. We performed the k-nearest neighbors’ imputation to fill in missing values for each fold^49^. We repeated the cross-validation procedure ten times to ensure robust results, each time using a different random number generator seed.

#### SMOTE

When a dataset is unbalanced, the feature selection and classification models frequently overestimate the likelihood of the majority outcome. As a result, the model may be inaccurate. We employed Synthetic Minority Oversampling (SMOTE) technique to increase model accuracy by balancing our unbalanced dataset. SMOTE accomplishes this by identifying the k-nearest neighbors (we chose k = 5 based on empirical evidence) and randomly generating new data along the line connecting two neighbors of the same class^50^. To ensure our dataset was balanced, we used SMOTE to oversample the number of cases for the less frequent outcome. We performed our analysis with and without SMOTE to determine whether it improved them and then reported the balanced dataset results.

### Statistical analyses

#### Logistic regression

All statistical analyses were performed using R^51^. We performed logistic regression analysis on each feature individually, keeping one-hot encoded features grouped together for the regression. After this univariate analysis, we performed multivariate logistic regression on all the features. Due to the high dimensionality of the dataset, we observed overfitting of the initial model. As a result, we performed multivariate logistic regressions using Akaike Information Criterion (AIC) and Bayesian Information Criterion (BIC), employing a sequential replacement method to identify subsets of features with low multicollinearity and strong association with the target variable^52^. We conducted these analyses using the *RcmdrMisc* library in R (RcmdrMisc). Along with AIC and BIC, we used the results of machine learning based feature selection algorithms to identify robust features for subsequent multivariate analysis as described above. Due to unplanned pairwise comparisons between features, p-values from the regression analysis were adjusted using the Benjamini–Hochberg method^53^.

#### Mediation analyses

We used mediation analysis to determine whether MEQ scores were statistically significant mediators between genotypic and clinical factors and mood disorders. The mediation’s significance was found using the R package *mediate*, which employs a nonparametric bootstrapping method to compute a confidence interval for the mediatory effects^54^.

#### Fisher’s exact tests

Fisher’s exact tests were used to identify features with a strong association with human anxiety individually. We created heatmaps illustrating patterns of association between molecular chronotype and human anxiety using different phase, amplitude, and STAI cutoffs.

#### Analysis of variance

To identify sex-specific differences in the average STAI scores for different two-way gene combinations, we used the *car* library in R to conduct a Type-3 Sum of Squares two-way ANOVA^55^. Tukey’s follow-up tests on significant factors were performed using the *emmeans* library. The normality of data was assessed by visual inspection and Shapiro–Wilk’s test in R.

#### Association rule learning analyses

We performed association analysis using the *arules* package^56^. The probability that an association occurs in the dataset is called its support. The lift of a rule is defined as the ratio of the observed support to that expected if the left-hand side and right-hand side of the relationship were independent. Since we had eleven variables, it was computationally intractable to find rules of length up to eleven with suitable support and lift. We limited ourselves to rules of size at most six, with at least 90% confidence. For each sex, we found rules that code for both categories of the target variable and then sorted them by their respective lift values. We visualized these relationships in sex-specific network plots, using the *igraph* library^57^.

#### Gene networks using mutual information

We employed the Algorithm for the Reconstruction of Gene Regulatory Networks (ARACNE) to find the direct and indirect interactions between genes, clinical features, MEQ, and mood disorders^58^. ARACNE constructs a network of relationships between nodes using a distance metric such as mutual information and correlation; for each triplet of edges, drops the edge with the lowest value. We used the *minet* package in R to create the network and then plotted it using *Rgraphviz*^59^ We used bootstrapping to determine the frequency (i.e., confidence level) with which each link in the network appears.

## Results

### Synergistic, two-way genotype combinations are predictive of human anxiety

**Figure 1:**
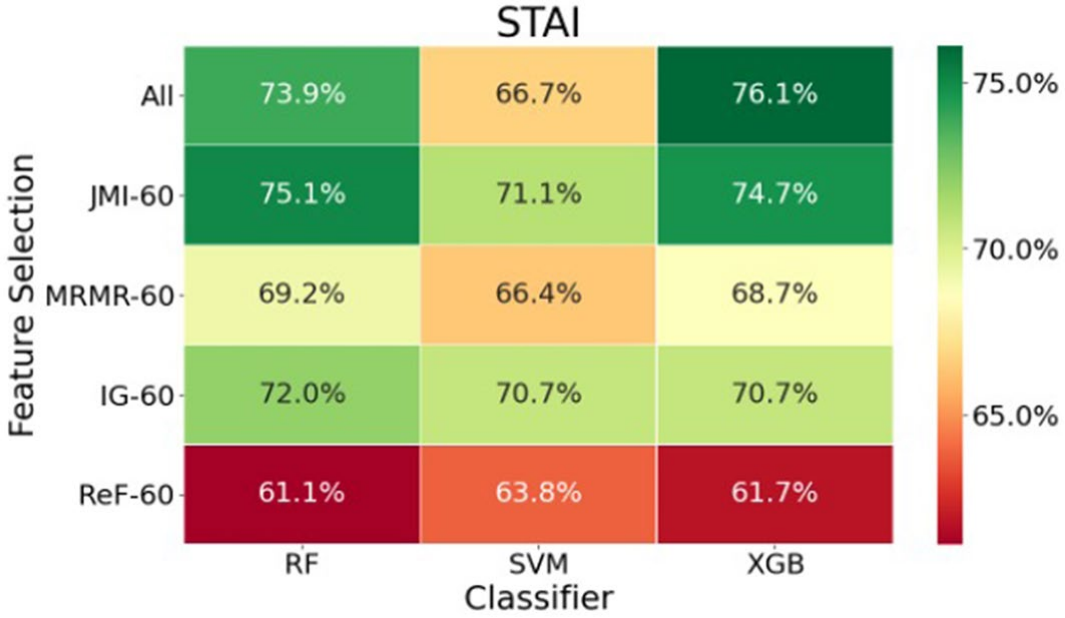
Heat map of prediction accuracy for feature selection and classifier methods. Our analyses yielded up to 26% higher prediction accuracy than baseline (50%) on a balanced data set.

RF, SVM, and XGB classifiers predicted anxiety symptoms with an accuracy of 61–76% using all or a subset of genotypic and clinical factors chosen by feature selection methods (Fig. 1). The XGB method achieves the highest accuracy (76%) when all features are used. However, if sixty features selected using the JMI method are used, a similar accuracy level (75%) can also be obtained using XGB and RF classifiers. These accuracy levels are 25%-26% more accurate than random chance in our balanced dataset, which has a baseline accuracy of 50%.

**Figure 2:**
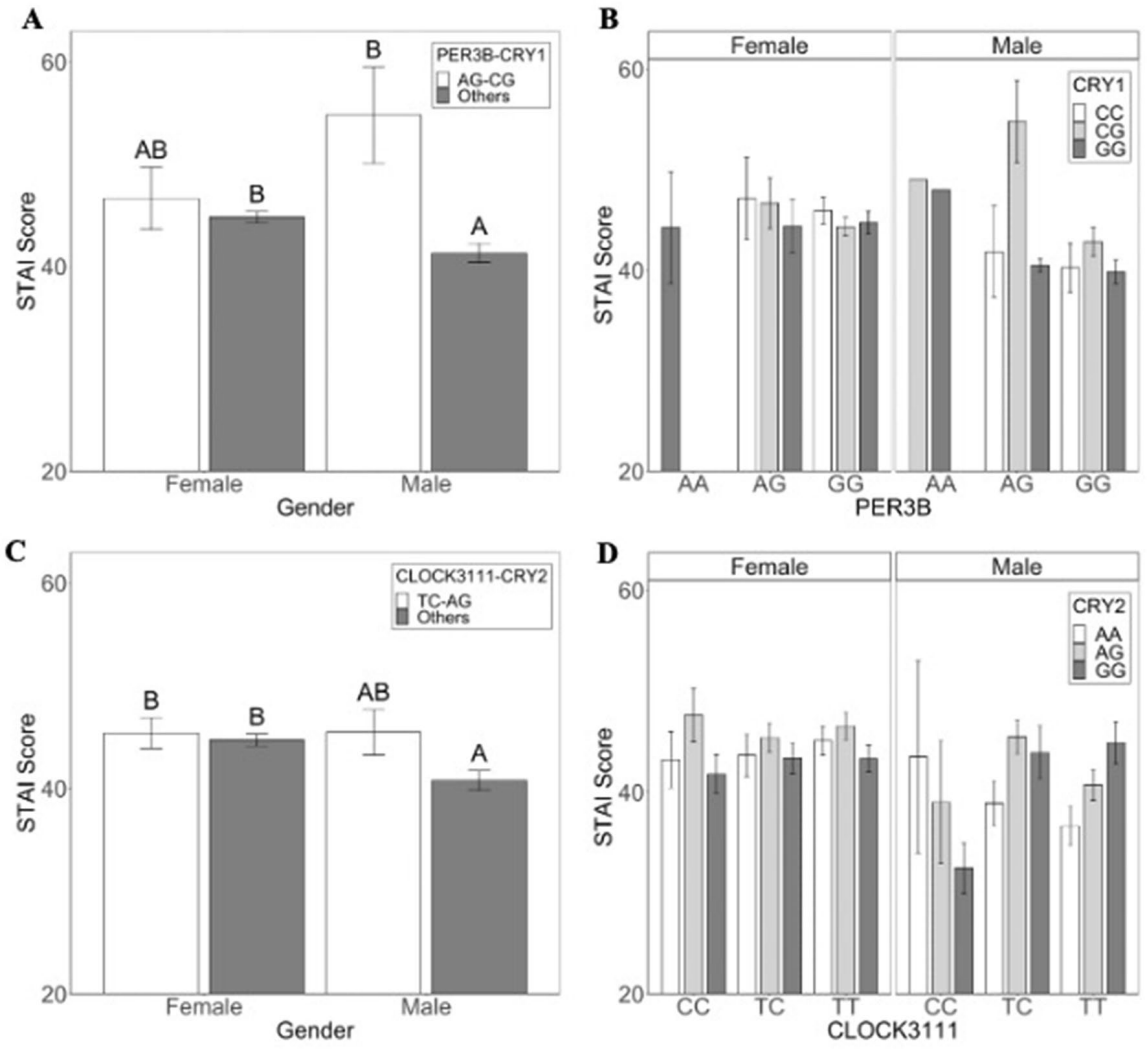
Genotype combinations predictive of anxiety symptoms in males. (**A,B**) Average anxiety scores for males with a combination of *PER3B*-AG and *CRY1*-CG are higher than average anxiety scores of individuals with other genotype combinations (Gender: F_1, 479_ = 0.661, p = 0.417, Genotype: F_1, 479_ = 7.174, p = 0.008; Gender x Genotype: F_1, 479_ = 4.141, p = 0.042). Anxiety scores are measured using the self-reported State-Trait Anxiety Index (± 1 SE). Tukey’s posthoc tests showed that males with AC-CG combination were significantly different from males with other genotypes and from females. (**C,D**) Average anxiety scores of males with a combination of *CLOCK*3111-TC and *CRY2*-AG also tend to be higher than average anxiety scores of individuals with other genotype combinations, but this is not significant at p < 0.05. (Gender: F_1, 517_ = 1.773; p = 0.184 , Genotype:F_1, 517_ = 3.417; p = 0.065; Gender x Genotype: F_1, 517_ = 1.912, p = 0.167).

Multivariate logistic regression analysis of the top features revealed that two-genotype combinations predicted a more robust risk of anxiety symptoms relative to single gene variants (Table 1). In the overall dataset, the combination of *PER3B*-AG and *CRY1*-CG was most strongly associated with the risk of having anxiety symptoms (Fig. 2; OR 15.3, p = 0.026). Average anxiety scores for individuals with *PER3B*-AG and *CRY1*-CG genotypes were higher (males: 54.8 ± 4.1; females: 46.7 ± 2.5) than for individuals of other genotypes (males: 41.3 ± 0.8, females: 44.8 ± 0.6; Fig. 2A,B).

**Table 1:** Risk factors for anxiety symptoms.

Variable	Odds ratio	95% CI	Adj P
PER3B_AG/CRY1_CG	15.262	2.352–319.019	0.026
PER3B_AG/CRY2_AA	0.17	0.034–0.694	0.026
AGE	3.851	1.234–14.006	0.031
CLOCK3111_TC/CRY2_AG	2.524	1.226–5.761	0.026
CRY1_CC/PER3_VNTR_4,4	2.432	1.029–6.781	0.061
GENDER	1.826	1.121–2.972	0.026
MEQ	0.958	0.935–0.982	0.005

Results from a multivariate logistic regression model based on top-ranked selected features identify two-way gene combinations, most notably *PER3B*-AG/*CRY1*-CG, as well as demographic features that are strongly associated with anxiety symptoms. Age is coded as 1 for 18–22 years old and 0 for > 22 years old. Gender is coded as 1 for females, and 0 for males.

The combination of *CLOCK*3111-TC and *CRY2*-AG also significantly increased the odds of anxiety symptoms (Fig. 2; OR 2.5 (1.3–5.8), p = 0.026). Average anxiety scores for individuals with *CLOCK*3111-TC and *CRY2*-AG were higher (males: 45.5 ± 1.7; females: 45.4 ± 1.5) than for individuals of other genotypes (males: 40.8 ± 0.9, females: 44.7 ± 0.6; Fig. 2C,D).

Additionally, the combination of *PER3B*-AG and *CRY2*-AA was protective against anxiety (Table 1; OR 0.17 (0.04–0.69), p = 0.026). Clinical features are also predictive of anxiety symptoms; females and young adults had a significantly higher risk of reporting anxiety symptoms (Table 1; age: OR3.9 (1.2–14.0), p = 0.026; gender: OR 1.8 (1.1–3.0), p = 0.026). Preference for morningness had a slight protective effect on the odds of reporting anxiety symptoms (OR 0.96, p = 0.005).

**Figure 3:**
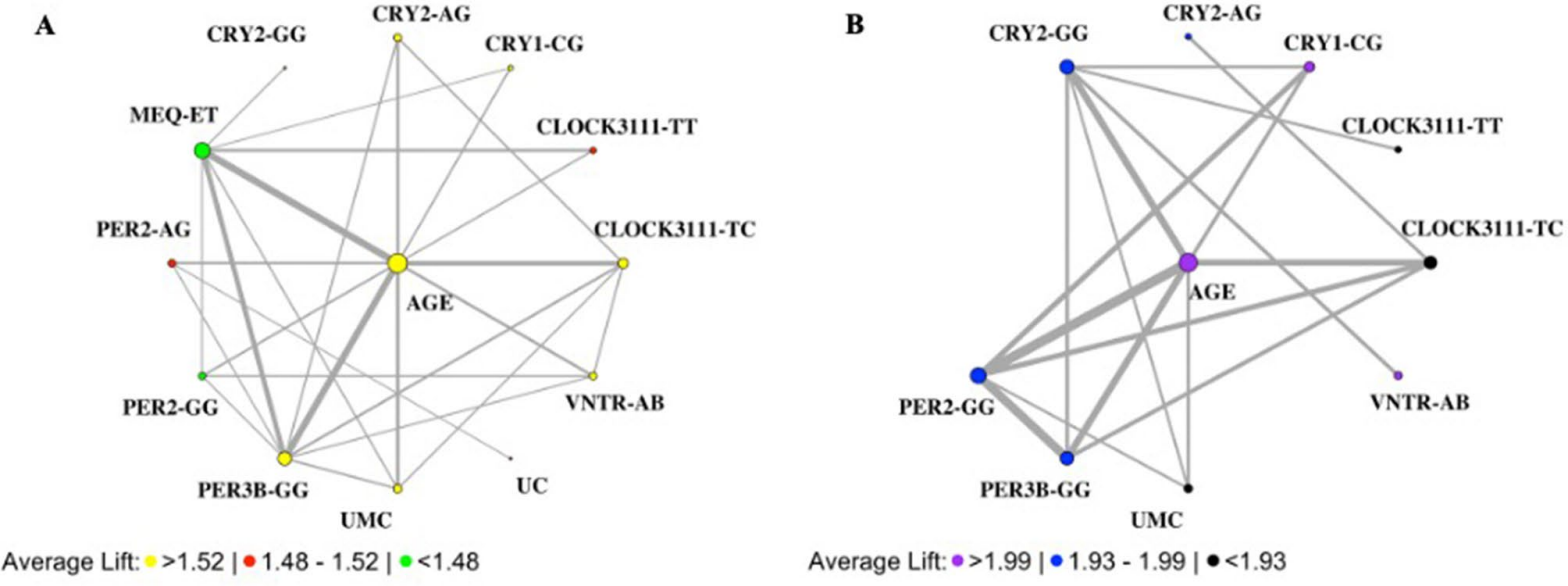
Association rules networks for anxiety symptoms. (**A**) In females, diurnal preference (MEQ), age, and *PER3B*GG co-occurred most frequently and had the highest average lift in the analysis. (**B**) In males, age, *PER2*-GG, and *PER3B*-GG co-occurred most frequently, but combinations with *CLOCK*3111TC had the highest average lift.

The association rule learning results identified additional multi-way genotype and clinical feature combinations that were strong predictors of human anxiety (Suppl. Table 2); risk genotypes differed for males and females (Fig. 3). For females, the most frequently appearing SNP variants in the top predictors of anxiety were *CLOCK*3111-TC and *PER3B*-GG, with age and MEQ as important clinical factors (Fig. 3A). For males, the most frequently occurring variants were *PER2*-GG, *PER3B*-GG, *CRY2*-GG, and *CLOCK*3111-TC, with age, but not MEQ, significantly predicting the risk of anxiety (Fig. 3B).

### Genotypic associations with anxiety symptoms can be direct or mediated by diurnal preference

Our network analysis using ARACNE on the interactions between genotypes, clinical features, and anxiety symptoms show that only the *PER3B* SNP variant (rs17031614) shares mutual information with anxiety symptoms via diurnal preference scores, which acts as a mediator (Suppl. Fig. 1). All other variants (*PER2*, *PER3* VNTR, *PER3A*, *CLOCK*3111, *CRY1*, and *CRY2* SNPs) were directly associated with anxiety symptoms following bootstrap analysis. The most robust links to anxiety symptoms are diurnal preference scores and depressive symptoms; the latter is likely due to the well-known co-morbidity of anxiety and depressive symptoms.

To further investigate the effects of MEQ on anxiety symptoms, we performed a mediation analysis of the top features. We found that the association between being a college-aged student and anxiety scores was mediated by diurnal preference scores. We also found that *PER2*-GG was strongly associated with an increase in anxiety scores (coefficient = 2.17, t_418_ = 2.09, p = 0.038) and the combination of *PER3B*-AG and *CRY2*-AG was weakly associated with anxiety scores (coefficient = 4.44, t_418_ = 1.85, p = 0.065); interestingly, these effects were completely mediated by diurnal preference score.

### Circadian phase, amplitude, and misalignment are associated with anxiety

**Figure 4:**
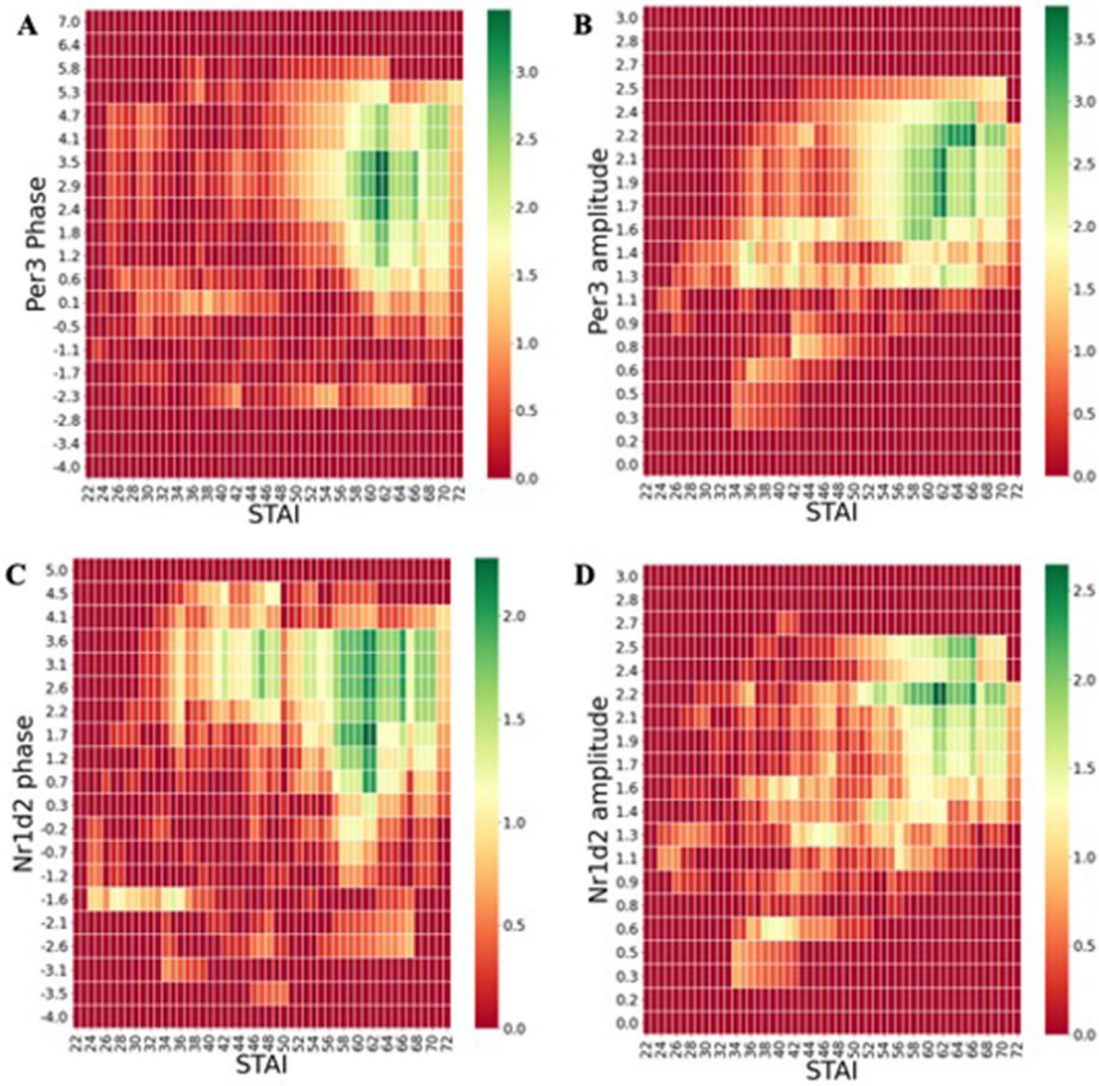
Heat maps of anxiety scores and circadian phenotypes. (**A,B**) Higher anxiety scores indicative of severe anxiety are strongly associated with positive *PER3* phase (advanced circadian phase or morning-types) and high circadian amplitude. (**C,D**) Similar patterns are seen with circadian phenotypes measured using the NR1D2 phase and amplitude. The heatmaps were made using Fisher’s Exact Test at varying cutoff levels for both dependent and independent variables. The p-values obtained from the analysis were log-transformed (base 10).

Using Fisher’s exact tests to test for significant associations across circadian phase and anxiety symptom scores, we found that advanced circadian phase values measured using a *PER3* or *NR1D2* gene markers (> 2.3 and > 1.7 standard deviations above the mean, respectively) were strongly associated with high anxiety scores. Similarly, high circadian amplitudes (> 1.7 and > 2.2 standard deviations above the mean, respectively) were also strongly associated with high anxiety scores. The detailed patterns of association are shown via heatmaps (Fig. 4).

Using gene expression data to measure the degree of mismatch in phase and amplitude with self-reported chronotype, we estimated the risk of anxiety symptoms with circadian misalignment. We found that individuals with advanced circadian phase and evening preferences (low MEQ scores) are six times more likely to report anxiety (*PER3*: OR 5.88 (1.22–28.40), p = 0.027; Nr1d2: OR 6.50 (0.77–58.48), p = 0.085).

## Discussion

A growing body of evidence indicates that alterations in circadian rhythms and clock gene mutations influence mood disorders, including anxiety. The search for molecular mechanisms underlying these connections has focused on GWAS and PheWAS studies. Still, these efforts are limited by the difficulty of predicting complex disorders with weak phenotyping and the inability to detect synergistic effects among genotypes. Further, identifying genotypes significantly associated with anxiety provides insufficient information to discern whether gene variants influence symptoms directly or indirectly, impeding the utility of this information for therapeutic interventions. This study uses a novel approach based on machine learning and statistical analysis to explore associations between circadian genes and anxiety symptoms in a deeply-phenotyped population sample. We report three findings: (1) synergistic interactions between variants in *PER3B*, *CLOCK*3111, and cryptochrome genes, *CRY1* and *CRY2*, show robust associations with anxiety symptoms, (2) clock variants predictive of anxiety symptoms tend to have sex-specific effects, and (3) molecular chronotype (circadian phase) and circadian misalignment—particularly individuals with advanced phases and evening-type sleep–wake cycles—are strong predictors of anxiety symptoms. Our results suggest that circadian clock gene variants have both direct (sex-specific) and indirect (clock-mediated) effects on anxiety symptoms.

### Circadian genotypes most predictive of anxiety

Our results confirm previous associations of anxiety with gender and chronotype and reveal novel associations of anxiety symptoms with circadian genotypes. We found that the combination of *PER3B*-AG and *CRY1*-CG was most strongly associated with the risk of having anxiety symptoms. The *PER3* gene encodes the period circadian protein homolog 3 protein in humans and is a paralog to the *PER1* and *PER2* genes. *PER3* is not essential for maintaining the circadian rhythm but plays a vital role in sleep–wake timing and sleep homeostasis. This gene is upregulated by CLOCK/BMAL heterodimers but then is repressed in a feedback loop involving PER/CRY heterodimers via interactions with CLOCK/BMAL complex. Previous studies have linked multiple SNPs and VNTRs in *PER3* to diurnal preference^7,17,23,24,27^. Archer et al.^18^ demonstrated a strong correlation between the extreme diurnal preference and the *PER3* variable number of tandem repeat (VNTR) polymorphism (rs57875989), with the longer allele associated with morning types and the shorter allele associated with evening types and delayed sleep phase syndrome in individuals. Liberman et al.^7^ also found that the *PER3* SNP (rs228697) was significantly associated with diurnal preference and anxiety symptoms.

In mammals, the *CRY* genes act as light-independent inhibitors of the CLOCK/BMAL heterodimers, which work as activators in the main circadian core loop^60^, and these genes also operate in the retina^61^. The *CRY1* variant (rs2287161) is an intergenic SNP located downstream from the gene’s 3′ polyadenylation site, suggesting that additional regulatory elements must aid in the modulation of *CRY1* gene expression. This variant has a robust association with depression in diverse populations^13,29,62^.

By what mechanisms do variants in *PER3*/*CRY* genotype combinations influence mood? *PER3* is upregulated by CLOCK/BMAL heterodimers but then represses this upregulation in a feedback loop using PER/CRY heterodimers to interact with CLOCK/BMAL. Variants in *PER3* and *CRY* genes may affect the dimerization of the proteins and the speed or success of the binding interaction with the CLOCK/BMAL complex, thus altering the sleep–wake cycle and influencing the timing of molecular pathways regulating mood. Alternatively, changes in PER3/CRY binding could affect the regulation of downstream pathways that directly influence mood pathways. This highlights a potentially critical role of CRY protein binding in modulating mood pathways.

Combinations of CLOCK3111/CRY2 variants may act similarly to the PER3B/CRY1 complexes, inhibiting the phosphorylation of the BMAL1/CLOCK dimer. In humans, *CLOCK* variants have been identified as important for both seasonal affective disorder (SAD) and bipolar disorder^8,15^. The *CLOCK*3111 SNP (rs1801260) is also linked to diurnal preference; individuals homozygous for the C allele have a stronger evening preference^63^. In non-human models, Roybal et al.^4^ show that ClockΔ19 mice exhibit hyperactivity, reduced anxiety- and depressive-related behavior, exhibiting a strong manic-like phenotype during the daytime. Transfections of mice with the *CLOCK*3111 variants revealed that the C-allele results in increased mRNA levels and stability^8^. In humans, Lavebratt et al.^64^ observed a significant association between the *CRY2* haplotypes with winter depression in Northern European populations. Furthermore, depressed bipolar patients have reduced levels of *CRY2*. Results from these previous studies indicate that both *CLOCK* and *CRY2* are associated with diurnal preference and can be a risk factor for depression and/or anxiety, suggesting that variants in these genes on mood may be direct or indirect effects. Our results suggest that the *CLOCK*3111 variant may directly affect anxiety symptoms, particularly in males, when found in combinations with the *CRY2*-AG variant.

### Epistatic effects

Complex traits with a polygenic basis, such as anxiety, may be likely to develop if the additive effects exceed a critical threshold that disrupts circadian rhythms^3,11^. In the current study, individuals who carry multiple circadian SNPs have an increased risk of anxiety symptoms relative to individuals who carry a single gene variant. Using feature selection methods, we can identify many two-genotype combinations that provide more significant effects on anxiety symptoms relative to the impacts of single SNP variants. Our association analysis allows for a more expansive exploration of multiple genes combinations. In these analyses, the top twelve rules with the strongest effects on anxiety symptoms included clinical features and two, three, and four-way gene combinations as strong predictors of human anxiety; only one factor represented a single gene (the *PER3* VNTR_5,5 genotype; Suppl. Table 1). The most frequently appearing SNP genotype in the association analysis was *CLOCK*3111-TC. Two of the rules with the highest lift values show the combination of *CLOCK*3111-TC, *CRY2*-AG, and *PER2*-AG as a significant predictor of anxiety. The combination of the first two SNP variants was identified as statistically significant by feature selection and logistic regression as well, but the additional additive effects of *PER2*-AG were identified by association rule learning. These findings imply that synergistic effects in the molecular clock are critical for modulating physiological pathways associated with anxiety.

### Potential direct effects on anxiety: sex-specific circadian genotype effects

One clue to which pathways are influenced by disruptions in the function of PER3B/CRY and CLOCK3111/CRY complexes is the fact that the effects of these variants on anxiety symptoms may be sex-specific. Previously, Shi et al.^65^ identified significant sex-dependent associations between major depressive disorder (MDD) and common variants of the circadian clock genes *CLOCK*, *PER3*, and *NPAS2*^6^. In that study, the association of *CLOCK* with MDD is also stronger in males, but the association of *PER3* and *NPAS2* with MDD is more significant in females. They propose that these SNPs have a functional effect via output transcriptional pathways that are mediated sex-dependently by the circadian system rather than the core clock oscillator^65^. One possible mechanism is glucocorticoid regulation, given that males and females have different cortisol levels^66^. Sex-specific, glucocorticoid-mediated stress responses may represent a mechanism by which clock genes affect anxiety and other mood disorders^67^. Other targets of *CLOCK*-mediated transcription involve neuropeptides and neurotransmitters, as well as their receptors, that may act to modulate mood pathways, including serotonergic pathways.

Interestingly, our network analyses also showed clear differences in key genotypic risk factors for males and females. The top rules for females included strong predictions for anxiety with *CLOCK*3111-TC, *PER3B*-GG, age, and MEQ, as well as weaker associations for other genotypes. Average lifts were higher for all of the top rules for males, but co-occurrence was more widely distributed across the genotypes, suggesting stronger associations between multiple gene variants and anxiety. In males, age, *PER2*-GG, *PER3B*-GG, and *CRY2*-GG co-occurred most frequently, but combinations with *CLOCK*3111TC had the highest average lift. Overall, our results suggest that the sex-specific anxiety risk conferred by the genotypic combinations involving *PER3B*, *CLOCK*3111, and *CRY2* genes may be further evidence of direct effects of clock gene binding complexes on downstream mood-related physiological pathways.

### Potential indirect effects on anxiety: mediation by diurnal preference and circadian misalignment

Following bootstrap analysis of the ARACNE gene network, *PER3B* was the only polymorphism in the current study with effects on anxiety that were significantly mediated by diurnal preference. In our targeted mediation analyses, associations of anxiety symptoms in *PER2* homozygotes for the G-allele were also significantly mediated by diurnal preference. This suggests that the Period family of genes may mediate mood via indirect pathways associated with circadian phenotypes and/or circadian misalignment.

Our molecular chronotyping results provide the strongest support for the indirect role of circadian clocks on mood by linking advanced phase, robust rhythms, and circadian misalignment to high levels of anxiety symptoms. Previous studies have shown that advanced *PER3* phase is strongly associated with morning types, while delayed phases values are more commonly found in evening types. In the current study, we show that higher *PER3* phase values were strongly associated with high human anxiety scores. Similarly, a higher *PER3* amplitude was also strongly associated with high anxiety. One potential explanation for these results is that individuals with stronger, more robust circadian amplitudes tend to have more robust sleep/wake patterns. Given that a large proportion of our study population consisted of undergraduates, the altered (i.e., typically delayed) sleep/wake patterns of college life may cause significant circadian misalignment, leading to high anxiety. To test whether the pattern of advanced phase and robust rhythms with high anxiety indicated the effects of circadian misalignment on mood, we identified individuals in our dataset with advanced circadian phases but who also reported evening-type patterns of sleep–wake behavior. The risk of anxiety symptoms was six times higher for these misaligned individuals, regardless of genotype, indicating that chronic disruptions to endogenous sleep–wake patterns increase anxiety symptoms in humans. A similar result was found for depressive symptoms in the same population^16^, suggesting that shifts in circadian circuitry may influence parallel pathways affecting both depressive and anxious symptoms.

### Limitations

This study should be viewed in the context of several limitations. Our machine learning and statistical analysis examined a large number of features for the relatively small sample size of the population. In addition, our population of primarily Caucasians of European descent limits the generalizability of our findings to diverse populations. We demonstrated that we could accurately predict anxiety using classification with a subset of features selected via feature selection. However, we are unable to quantify the classification prediction accuracy using only the top robust features since we used the entire data set (due to the small sample size) to attain these features. Our estimation of delayed or advanced circadian phase utilized an analysis of gene expression from two genes at four data points; greater accuracy in estimation may be achieved with analysis of additional genes or data points. Future studies should assess the accuracy of our anxiety risk factor predictions using an independent population sample. Finally, our statistical analyses report significant differences in anxiety risk associated with particular genotypes and circadian phenotypes; further functional and behavioral studies are needed to understand how therapeutic targets or behavioral interventions might be designed to mitigate anxiety symptoms in humans.

## Conclusion

Here, we report both direct and indirect, via mediation by circadian phenotypes, effects of circadian genotypic and clinical features on anxiety symptoms. Using an approach that employs machine learning and statistical analyses to examine associations of circadian clock genes with human anxiety, our results support three conclusions. First, variants in select circadian clock genes have synergistic associations with anxiety symptoms. Second, sex-linked associations between clock gene variants and anxiety symptoms provide evidence of multiple direct pathways for clock genes to influence mood. Finally, molecular chronotype and circadian misalignment are strong predictors of anxiety symptoms, indicating that indirect effects of clock gene variants, particularly in the *PER3* gene, may also play a role in modulating anxiety symptoms. Disentangling the complex influences of clock genes on anxiety may reveal both clinical targets and non-invasive therapies that can help mitigate the causes and symptoms of anxiety.

## Supplementary Information

Supplementary Table 1.

Supplementary Table 2.

Supplementary Figure 1.
