# Supplementary material for: Machine learning and expression analyses reveal circadian clock features predictive of anxiety
Source: Sci Rep. 2022 Apr 1;12:5508. doi: 10.1038/s41598-022-09421-4 (PMC8975926; doi:10.1038/s41598-022-09421-4)
Supplement: Supplementary file 1 — Supplementary Table 1. [file 41598_2022_9421_MOESM1_ESM.docx]

**Suppl. Table 1. Circadian gene variants and proposed associations with chronotype/diurnal preference and anxiety and/or depression.**

|  | | |  |  | **Proposed Mechanisms in Literature** | | **Proposed Mechanisms in Current Study** | | |
| --- | --- | --- | --- | --- | --- | --- | --- | --- | --- |
| **Gene** | ***rs#*** | ***Genotype*** | ***MAF*** | ***Mutation*** | ***Direct*** | ***Indirect*** | ***Direct*** | ***Indirect*** | ***Unknown*** |
| CLOCK | ﻿  rs1801260 | T/C | 0.25 | 3’UTR | C-allele in males with MDD (Shi et al., 2016) | C-allele associated with delayed phase and evening chronotype (Katzenburg et al., 1998); association with seasonal depression (Kim et al., 2015) | TC genotype increased risk of anxiety in males |  |  |
| CRY1 | ﻿rs2287161 | C/G | 0.45 | Upstream of promotor | C-allele and CC genotype associated with depression (Soria et al., 2010; Hua et al, 2014; Liberman et al., 2018) |  | CG genotype increased risk of anxiety in males |  |  |
| CRY2 | rs10838524 | A/G | 0.53 | Intron |  | G-allele associated with SAD (Lavebratt, et al., 2010b) and eveningness (Liberman et al, 2018) | AG genotype increased risk of anxiety in males |  | AA genotype protective against risk of anxiety |
| PER2 | ﻿rs10838524 | G/A | 0.4 | Intron | G-allele with MDD (Lavebratt et al., 2010a) |  |  | GG-genotype  association with anxiety mediated by MEQ score |  |
| PER3 VNTR | ﻿rs57875989 | PER3^4,4^  PER3^4,5^  PER3^5,5^ | 0.12 | In frame deletion of the repeat region | 4 allele with anxiety  (Liberman et al., 2017); 4,4 genotype with anxiety & depression (Viena et al., 2016; Liberman et al., 2018; Silva et al. 2020) | 4,4 genotype with evening chronotype  (Archer et al., 2003; Ebisawa et al., 2001; Hida et al. 2014; Liberman et al, 2017, 2018) |  | High mPer3 phase and amplitude with anxiety | 5,5 genotype with anxiety |
| PER3A | ﻿rs228697 | C/G | 0.08 | missense Pro856Ala | GG genotype with MDD and anxiety (Liberman et al., 2017, 2018; Shi et al., 2016) | G-allele and GG genotype with eveningness ﻿(Hida et al., 2014; Liberman et al, 2018) |  | High mPer3 phase and amplitude with anxiety |  |
| PER3B | rs17031614 | G/A | 0.08 | Synonymous | A-allele with depression (Shi et al., 2016) |  | AG genotype increased risk of anxiety in males | PER3B association with anxiety mediated via MEQ scores |  |

*proposed mechanisms
