# Supplementary material for: Machine learning and expression analyses reveal circadian clock features predictive of anxiety
Source: Sci Rep. 2022 Apr 1;12:5508. doi: 10.1038/s41598-022-09421-4 (PMC8975926; doi:10.1038/s41598-022-09421-4)
Supplement: Supplementary file 2 — Supplementary Table 2. [file 41598_2022_9421_MOESM2_ESM.docx]

**Supplementary Table 2. Association Rules Predicting Anxiety Symptoms (STAI).**

| Rules | Support | Confidence | Coverage | Lift | Count |
| --- | --- | --- | --- | --- | --- |
| {GENDER,CRY2_GG,MEQ_ET} | 0.05 | 0.93 | 0.05 | 1.87 | 28 |
| {AGE,GENDER,CRY2_GG,MEQ_ET} | 0.05 | 0.93 | 0.05 | 1.87 | 28 |
| {GENDER,CRY2_GG,PER3B_GG,MEQ_ET} | 0.04 | 0.93 | 0.05 | 1.85 | 25 |
| {AGE,GENDER,CRY2_GG,PER3B_GG,MEQ_ET} | 0.04 | 0.93 | 0.05 | 1.85 | 25 |
| {AGE,CLOCK3111_TC,PER2_AG,CRY2_AG} | 0.04 | 0.92 | 0.04 | 1.84 | 23 |
| {GENDER,VNTR_4,5,MEQ_ET} | 0.05 | 0.90 | 0.05 | 1.80 | 27 |
| {AGE,GENDER,VNTR_4,5,MEQ_ET} | 0.05 | 0.90 | 0.05 | 1.80 | 27 |
| {GENDER,VNTR_4,5,PER3B_GG,MEQ_ET} | 0.04 | 0.89 | 0.05 | 1.78 | 24 |
| {AGE,GENDER,VNTR_4,5,PER3B_GG,MEQ_ET} | 0.04 | 0.89 | 0.05 | 1.78 | 24 |
| {CLOCK3111_TC,PER2_AG,CRY2_AG} | 0.04 | 0.88 | 0.05 | 1.77 | 23 |
| {AGE,VNTR_5,5,PER3B_GG} | 0.05 | 0.88 | 0.06 | 1.75 | 28 |
| {VNTR_4,5,CLOCK3111_TC,PER2_GG} | 0.05 | 0.87 | 0.05 | 1.74 | 27 |
| {AGE,VNTR_4,5,CLOCK3111_TC,PER2_GG} | 0.05 | 0.87 | 0.05 | 1.74 | 27 |
| {AGE,CLOCK3111_TC,CRY2_AG,SOCIOSTATUS_3.0} | 0.04 | 0.86 | 0.05 | 1.71 | 24 |
| {AGE,CLOCK3111_TC,PER3B_GG,MEQ_ET} | 0.05 | 0.85 | 0.06 | 1.71 | 29 |
| {AGE,VNTR_4,5,CLOCK3111_TC,CRY2_AG} | 0.05 | 0.85 | 0.06 | 1.71 | 29 |
| {CLOCK3111_TC,PER2_GG,SOCIOSTATUS_3.0} | 0.04 | 0.85 | 0.05 | 1.70 | 23 |
| {VNTR_4,5,CLOCK3111_TC,PER2_GG,PER3B_GG} | 0.04 | 0.85 | 0.05 | 1.70 | 23 |
| {AGE,CLOCK3111_TC,PER2_GG,SOCIOSTATUS_3.0} | 0.04 | 0.85 | 0.05 | 1.70 | 23 |
| {AGE,VNTR_4,5,CLOCK3111_TC,PER2_GG,PER3B_GG} | 0.04 | 0.85 | 0.05 | 1.70 | 23 |
| {AGE,VNTR_5,5} | 0.05 | 0.85 | 0.06 | 1.70 | 28 |
| {GENDER,VNTR_4,5,PER2_GG,PER3B_GG} | 0.05 | 0.83 | 0.06 | 1.67 | 30 |
| {AGE,GENDER,VNTR_4,5,PER2_GG,PER3B_GG} | 0.05 | 0.83 | 0.06 | 1.67 | 30 |
| {AGE,GENDER,CLOCK3111_TC,PER2_AG,PER3B_GG} | 0.06 | 0.83 | 0.07 | 1.66 | 34 |
| {VNTR_5,5,PER3B_GG} | 0.05 | 0.83 | 0.06 | 1.66 | 29 |
