# Supplementary material for: Machine learning and expression analyses reveal circadian clock features predictive of anxiety
Source: Sci Rep. 2022 Apr 1;12:5508. doi: 10.1038/s41598-022-09421-4 (PMC8975926; doi:10.1038/s41598-022-09421-4)
Supplement: Supplementary file 3 — Supplementary Figure 1. [file 41598_2022_9421_MOESM3_ESM.docx]

**
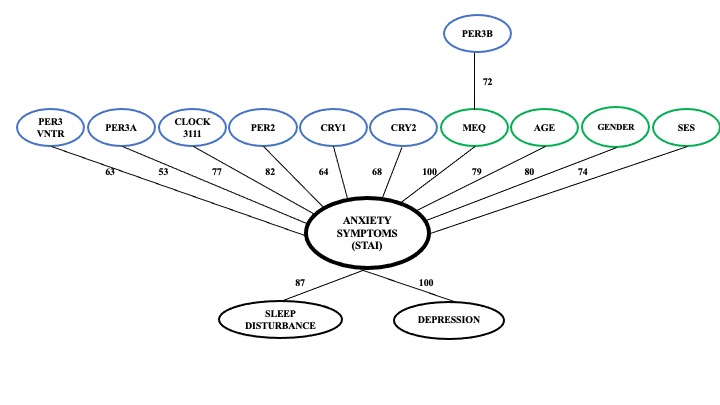
**

**Supplementary Figure 1. MEQ acts as a mediator between PER3B and human anxiety**. The network is constructed using the ARACNE method in conjunction with the mi.empirical method and bootstrapping. All links with bootstrap support greater than 50% are shown. The most frequently observed associations were between MEQ and anxiety, and between anxiety and depression. MEQ mediates the association between PER3B and anxiety, but not the association between anxiety and other genotypic factors. SES: Socio-economic status.
